# Extended LineSets: a visualization technique for the interactive inspection of biological pathways

**DOI:** 10.1186/1753-6561-9-S6-S4

**Published:** 2015-08-13

**Authors:** Francesco Paduano, Angus Graeme Forbes

**Affiliations:** 1 Department of Computer Science M/C 152, University of Illinois at Chicago, 851 S. Morgan, Room 1120, Chicago 60607-7053, IL, USA

**Keywords:** Pathway Visualization, Hierarchical Inspection, Eliminating Node Redundancy, Line Sets, Interactive Visual Analysis

## Abstract

**Background:**

Biologists make use of pathway visualization tools for a range of tasks, including investigating inter-pathway connectivity and retrieving details about biological entities and interactions. Some of these tasks require an understanding of the hierarchical nature of elements within the pathway or the ability to make comparisons between multiple pathways. We introduce a technique inspired by *LineSets *that enables biologists to fulfill these tasks more effectively.

**Results:**

We introduce a novel technique, *Extended LineSets*, to facilitate new explorations of biological pathways. Our technique incorporates intuitive graphical representations of different levels of information and includes a well-designed set of user interactions for selecting, filtering, and organizing biological pathway data gathered from multiple databases.

**Conclusions:**

Based on interviews with domain experts and an analysis of two use cases, we show that our technique provides functionality not currently enabled by current techniques, and moreover that it helps biologists to better understand both inter-pathway connectivity and the hierarchical structure of biological elements within the pathways.

## Background

Much effort has been expended to organize the body of knowledge that is available regarding the structure and function of biological pathways. The Reactome Database [1] and the KEGG Pathway Database [2] are just two examples of publicly accessible resources of biological data. These databases, and the frameworks created to access, process, and query them, such as *Pathways Commons *[3], allow biologists to investigate different pathways that may share common elements, such as biochemical reactions or protein complexes. The flexibility of these search tools, and the scale of the data that can be quickly retrieved, has motivated researchers to design new visualization tools to assist in a range of analysis tasks involving multiple pathways. A catalog of requirements for pathways visualization tools are detailed by Saraiya et al. [4], who stress the need of further research into interactive, dynamic solutions.

Pathways are typically represented as directed graphs, where nodes in the graph represent biological "participants," such as proteins or protein complexes, and where the edges represent a biological functionality, such as a biochemical reaction. Often different shapes for arrows and nodes are used to differentiate between the different types of molecules or reactions. Though this type of visual encoding is the most familiar, node-link diagrams are known to have a number of issues. A main issue is scalability; as the number of nodes or edges increases, it quickly becomes more difficult to make sense of the data [5]. In the last decade, analyses that involve thousands of proteins or genes have become conventional. Numerous attempts have been proposed to visualize and analyze large biological networks, with particular attention to the topology of the network and its hierarchical structure.

The importance of dynamic visualization has been discussed by Hu et al. [6] and Klukas and Schreiber [7]. Static images can depict a carefully arranged, fine-tuned representation that improves readability, but this advantage is exceeded by the navigation methods typically supported by dynamic visualizations. Furthermore, it is important that the layout can be programmatically changed by the user, and that additional components can be added to the existing depiction.

The structure of biological pathways can be formalized as a hypergraph, a graph that contains hyperedges which can connect to any number of nodes. Traditional node-link diagrams cannot provide sufficient information to represent all of the complexities in a biological pathway data: biochemical reactions can involve multi-participant relationships; pathways can contain multiple subpathways; nodes within a pathway can represent a nested structure containing many biological entities; and links between nodes can convey different biological meanings. Some techniques for the visualization of pathways propose representations which limit the complexity of the data structure in favor of a simpler design. This is the case of the SIF format, which is often used to encode data for generating visualizations with tools such as *Cytoscape *[8]. This format represents only binary relationships and excludes rich biological semantics. Other formats include a more complex description of biological interactions, and require more sophisticated visual representations that leverage user-driven interaction and innovative visual encodings.

This paper presents a novel technique, *Extended LineSets*, that more accurately represents the intricacy of interconnected pathways and subpathway connections, and moreover, that helps to reduce visual clutter that can interfere with visual analysis tasks. Additionally, we describe a prototype implementation that makes use of this technique so that biologists can more effectively search, filter, visualize, and compare pathways data.

### Task analysis

We interviewed seven domain experts in order to understand the type of tasks that could be usefully accelerated or augmented by visualization techniques. The experts are professors and researchers in different domains of cellular biology, molecular genetics, and informatics. While each of the experts have different research interests, we identified three high-level tasks that were considered to be important to all of them. Through defining these tasks, we were able to identify the primary requirements necessary for an effective visualization technique. These tasks are not meant to be comprehensive, but rather to provide insight into the motivation for the development and design of *Extended LineSets*.

**Task 1: Examine the upstream and downstream connections between two entities within a pathway**. Understanding how entities within pathways are connected is of critical importance to all of the researchers we interviewed, and is essential to most research related to pathway data. When discussing directed paths between entities, one entity is said to be *upstream *or *downstream *of another. Understanding upstream and downstream relationships is particularly important to domains such as cancer drug research, where a drug may affect a small subset of genes or gene products, which in turn will affect various downstream processes. In most cases, a directed relationship is meant to represent a biochemical reaction, where one entity is consumed as a reactant and another is produced as a product. Thus, an upstream entity may be connected to a downstream entity through a chain of several directed links. In the most basic sense, the "entities" mentioned above are genes, gene products (such as proteins or complexes), or other small molecules within a cell. A researcher may be interested in understanding the path of reactions (or other relationships) that connects two entities.

**Task 2: Understand the hierarchical structure of protein complexes**. Protein complexes are represented as nested hierarchies of proteins. These hierarchies can be very intricate and potentially involve dozens of proteins and sub-complexes. Since the proteins and the complexes which compose these compound structures might be involved in other relevant complexes and biological processes, understanding their structure and organization is generally important for pathway analysis tasks.

**Task 3: Curate, edit, query and merge pathway data files, or construct user-defined pathways**. Several of the researchers mentioned the importance of various tasks related to the curation, maintenance, and understanding of pathway data. They expressed the need to create "personalized pathways" that only include a user-determined subset of entities and relationships. They are especially interested in mixing-and-matching information that is contained within multiple pathways, but then being able to filter this information so that only information relevant for a particular task is displayed.

### Related work

It can be challenging to represent multiple pathways simultaneously in traditional node-link diagrams, due to issues of scalability and also due to difficulties in defining a layout that accurately and efficiently displays the topology of these pathways. In order to reduce the visual clutter introduced by dense networks with many edge crossings, many visualization tools rely on *node duplication*, which considerably reduces the overlapping of edges and enables a visually appealing arrangement of the nodes. However, as pointed out by Bourqui et al. [9], analysis tasks that rely on an understanding of the pathway topology might be hampered by the introduction of duplicated nodes.

This section presents an overview of features implemented in popular pathway visualization tools. One feature missing from all of these tools is a specific visualization technique for presenting category data within node-link diagrams. Indeed, as indicated by *Task 3*, researchers may need to merge pathway components from multiple sources, while still retaining an indication of the original source. For this reason, we also review visualization techniques that are used for presenting category information.

#### Pathway visualization tools

Broadly speaking, current tools make use of one of three primary visual representations: node-link diagrams, node-link diagrams with compound nodes, and adjacency matrices. *Entourage *[10], *Reactome Pathway Browser *[11], *VisAnt *[12], *MetaViz *[9] and *VitaPad *[13], each use a traditional node-link diagram as a primary visual representation. *ChiBe *[14] extends the node-link representation, additionally displaying compound nodes that indicate the composition of complexes. Rather than enabling an interactive exploration of the hierarchical structure of protein complexes, it instead depicts all sub-elements in a single node. This approach might lead to visual clutter when depicting complex nested structures. The tool has a "merge pathways" function that combines the visualization of multiple pathways in the same view. This approach is similar to our technique, but we provide a visual correspondence that emphasizes the origin of elements from particular pathways.

The *Reactome Pathway Browser *is a tool for visualizing pathway diagrams included in the *Reactome *database. It offers basic navigation and limited interactivity, making use of side panels to enable the inspection of complexes structure and analysis data. *Entourage *is a tool for pathway visualization that enables the exploration of the "cross-talk" between pathways via an "artificial partitioning" of the pathway structure, helping to reduce the complexity of the visualization. Multiple pathways and sub-pathways are represented in isolated views, which preserves the pathway structure but introduces node redundancy. Other tools represent multiple pathways in a single view, such as *MetaViz*, which enables topological analyses across multiple metabolic pathways simultaneously. Our work is inspired by *MetaViz*, but our technique tries to organize the representation of all pathways according to a topological ordering and enables the interactive inspection of hierarchical structures of certain nodes. *BioFabric *[15] also enables the exploration of large biological networks composed by multiple pathways without replicating nodes. This tool is meant to visualize extremely large networks with thousands of nodes. However, it was not specifically designed for visualizing pathways and it does not enable pathway-specific tasks. *VisAnt *enables the user to search for the shortest path between two nodes [16] and to identify dense, highly-connected nodes. The positions of the nodes in the graph are computed with a "relaxing layout" algorithm which models the network as a set of physical entities. This algorithm builds a graphical representation that organizes the graph by density of the connections between cluster of nodes. A similar approach has been adopted in this work. Furthermore, *VisAnt *permits a dynamic exploration of a biological network composed by multiple pathways. However, if more than one pathway includes the same node, multiple instances of the node will be present in the representation.

Although the majority of these pathway visualization tools use conventional graph representations, many of them decorate the node-link diagrams with experimental data or other additional information. For example, *VitaPad *allows the user to incorporate microarray data into pathways, *Entourage *enables the integration of experimental data, and *VisAnt *permits the user to create and view annotations of nodes within the network.

#### Visualizing categories on node-link diagrams

Combining multiple pathways in a single visualization introduces the need for displaying the relationships between biological entities and the pathway or pathways they are participants of. That is, biochemical reactions, proteins, or other biological compounds can be included in more than one pathway. This raises the further challenge of effectively displaying this membership information on top of the traditional node-link diagram without introducing visual clutter.

Dinkla et al. [17] propose a solution which integrates node-link diagrams with *Euler Diagrams *to display set-based annotations on biological networks. However, this may lead to visually confusing representations if the data contains complex set intersections. For this reason a range of alternative visualization techniques has been developed to display elements belonging to multiple sets [18]. *Bubble Sets *[19], *Line Sets *[20], and *KelpFusion *[21] all address this issue. Each of these techniques are designed to be overlaid on top of existing visualizations, such as geographical maps. Some aspects of both *Bubble Sets *and *KelpFusion *prevent these techniques from being applicable to pathway visualization. For example, *Bubble Sets *displays set relations using isocontours, which can make it difficult to distinguish elements belonging to multiple sets.

*LineSets*, developed by Alper et al. [20], is a technique that represents sets as smooth curves and uses colors to indicate membership. This solution offers better readability than the *Bubble Sets *technique when multiple sets overlap. *KelpFusion *uses continuous boundaries made by lines and hulls. The visual appearance is generally comparable to *LineSets*, but this strongly depends on the spatial arrangements of elements. Hulls are used to group elements that are both spatially close to each other and that belong to the same set. This technique can become confusing when it is applied to biological networks, which have an arbitrary or dynamic spatial layout. *LineSets*, though it has been applied to geographic datasets, is a more extensible technique that is able to effectively represent pathway data.

None of the above techniques scale well when the number of sets and number of intersections between sets increases. *UpSet *[22] and *OnSet *[23] are two recent visualization techniques designed to help users better understand complex relationships between a large number of sets. Although these sophisticated and scalable tools enable several set visualization tasks, they do not explicitly aim to integrate with node-link representations.

In this paper we introduce *Extended LineSets*, a modified version of the *LineSets *technique which introduces the inclusion of hierarchical structure and which is presented as a more abstract node-link diagram, without the need for elements to have a spatial reference. Our technique aims to enhance analysis tasks that make use pathway visualization tools. It adds an extra information layer to the node-link diagram that represents the intricate relationships within and between pathways, and at the same time it provides an approach that reduces clutter while avoiding node duplication. In the following section we include a detailed description of the *Extended LineSets *technique. After presenting a prototype implementation, we introduce two use cases that demonstrate how our technique enables effective, real-world pathway exploration.

## Methods

In this section we describe the two primary representations used in the *Extended LineSets *technique. The first representation combines node-link diagrams with *LineSets*, allowing the user to concurrently identify set memberships in a network and to visualize directional relationships of between elements. The second representation displays the hierarchical layout of elements within the network, allowing the user to interactively inspect their nested structure. These two primary representations are integrated to support the effective interactive investigation of biological pathways. Our prototype utilizes our technique and implements a variety of user-driven interaction to enable pathway analysis tasks.

### Extending node-link diagrams with *LineSets*

**Figure 1:**
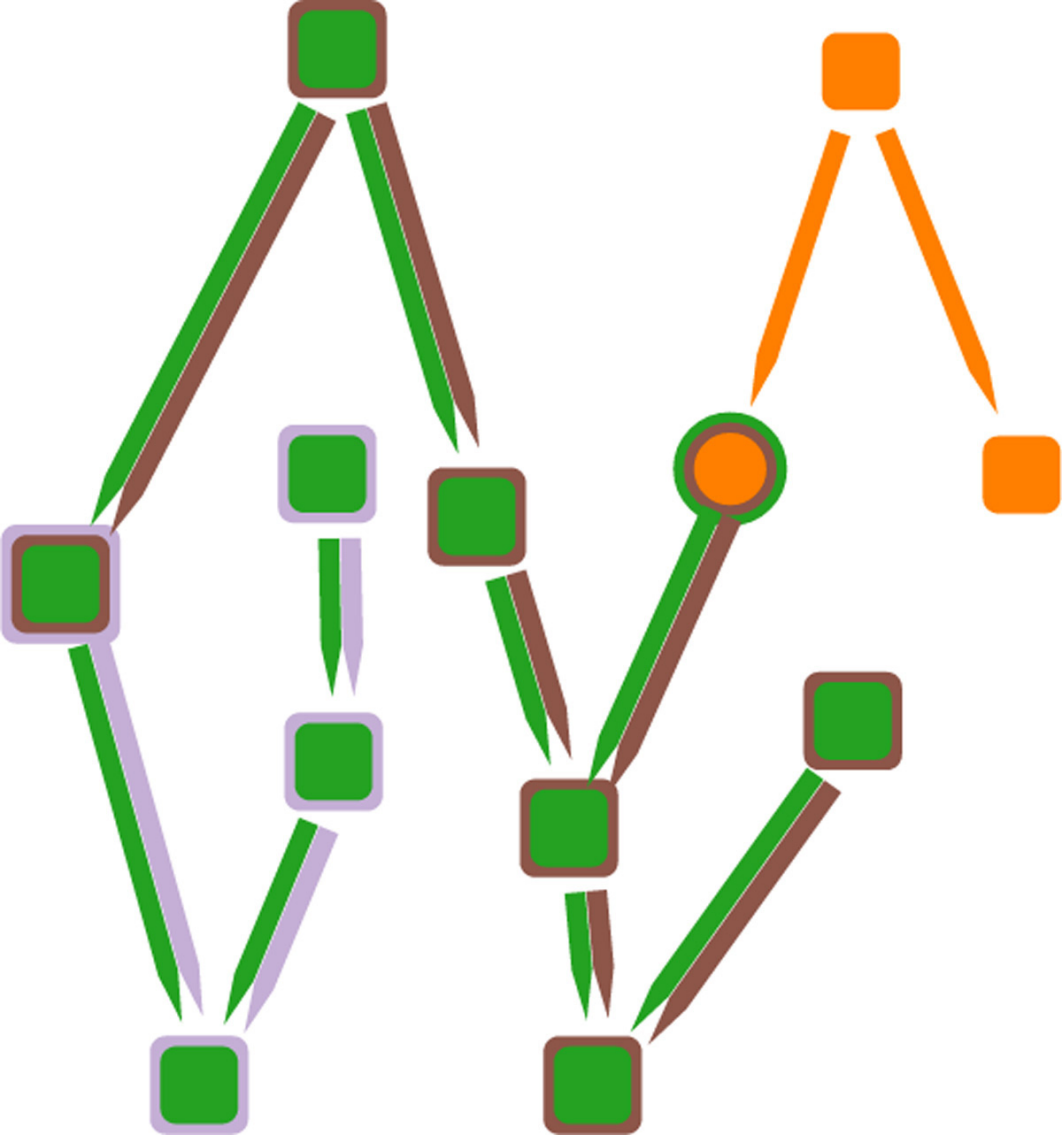
**The image shows the proposed technique to combine node-link diagram with *LineSets***. Colors are used to indicate set membership of nodes and links; the nodes are arranged to follow the topological ordering of the links; and the pointed edges indicate directionality.

Our first visual component presents a directed graph where nodes and edges belong to one or more sets. We identify every set with a color, and every element is depicted as either a square or a circle. Edges linking elements are represented using colored lines with sharp-pointed tip. Figure 1 shows an example of how nodes and links belonging to one or more sets are depicted using our technique. If the same node is included in more than one set, another colored border is added for each additional pathway it is associated with. Moreover, if more than one set includes an edge between the same elements, then multiple sharp-pointed line are placed side by side. The line direction and colors indicate the corresponding set and the direction of the edge. The segment connecting two components has a sharp tip on both extremities if two opposite edges in the same set involve the same elements.

**Figure 2:**
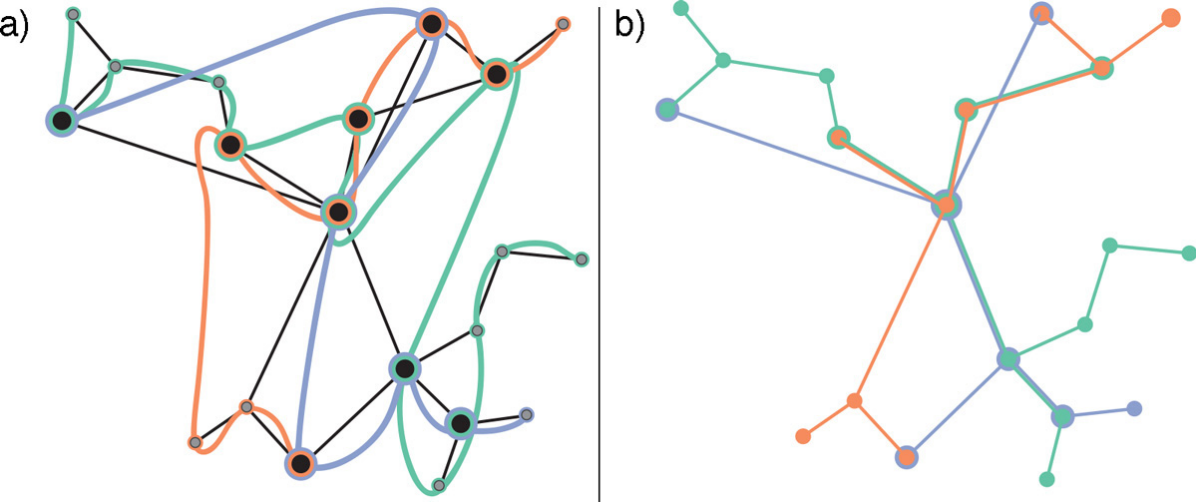
**The image compares the same dataset realized with (a) *LineSets*, where showing set information and network information simultaneously leads to visual clutter, and (b) *Extended LineSets*, which mitigates visual clutter via a more effective depiction of category information**.

This visualization component has some substantial differences from the original *LineSets *implementation. First, every set is not represented by a single smooth curve, but is identified only by the color of the links and nodes. Second, the layout of the elements flows from the top to the bottom, matching, when possible, the direction of the relationships. A completely consistent representation cannot be achieved when the graph contains cycles; in this case some directed edges will point upwards, but the general layout will still mostly adhere to the flow from the top to the bottom of the viewport. Additionally, the layout of the elements is designed to minimize nodes overlapping and edges crossing. The resulting visualization combines the efficient identification of elements belonging to particular sets from the *LineSets *technique with a traditional node-link graph representation. Figure 2 compares a small dataset realized using *LineSets *(Figure 2a) with the same dataset realized using our technique (Figure 2b).

**Figure 3:**
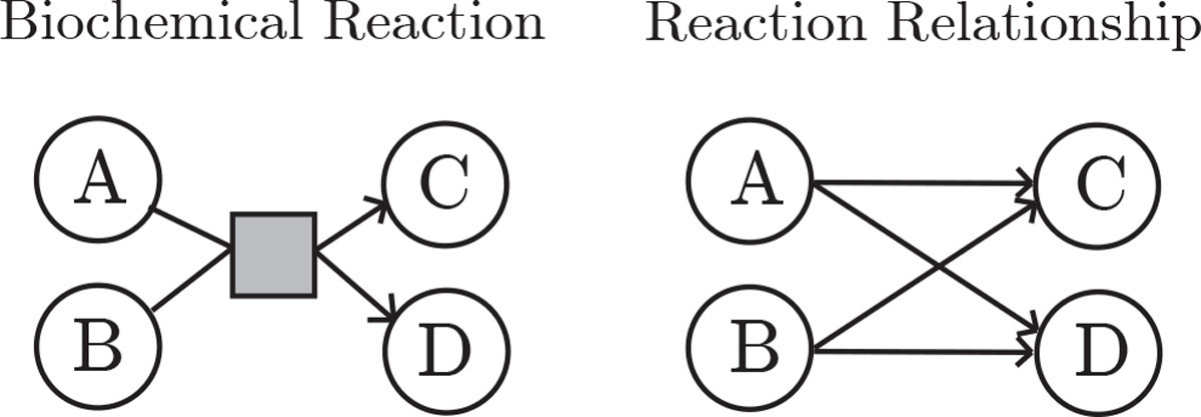
**On the left we depict a biochemical reaction involving two left components (A and B) and two right components (C and D)**. On the right we depict the "reaction relationship" between these four biological participants in our technique (both A and B link to C and D). That is, we encode the biochemical reaction as *links *between input and output elements.

Our technique avoids node duplications and effectively shows the relationships between different sets. Each individual pathway is considered a set and is assigned a unique color. The elements in the set include proteins, complexes, and links. The links between elements indicate *reaction relationships*. A reaction relationship exists between components *A *and *B *in the pathway *P *if *P *has at least one reaction in which the inputs include *A *and the outputs include *B*. Figure 3 provides an example of how a biochemical reaction that involves two input participants and two output participants is converted to four distinct directed edges, in accordance with the previous definition. Furthermore, each node is assigned either the shape of a circle or a square; circle-shaped nodes indicate proteins and square-shaped nodes indicate protein complexes.

### Hierarchical inspection

In order to support *Task 2*, a pathway visualization tool must enable the inspection of the structure of biological complexes. The inspection of hierarchical structures is supported by our interaction visualization technique. Using the traditional notation for tree structures, we refer to the outermost component of the structure as the *root *element, a *parent *is an element that contains *children *elements, and elements with no children are *leaves*. However, the conventional representations of hierarchical structures with traditional tree diagrams are prone to visual clutter as the amount of leaves and the depth of the tree increases [24].

We mitigate this issue through using two coordinated views: a *symbolic overview *and a *pruned tree schematic*. The symbolic overview indicates the overall structure of the protein complex using a rectangle packing layout, whereas the pruned tree schematic presents a more detailed view of the different levels in the hierarchy. No labeling is used in the symbolic overview in order to minimize visual clutter, but these details can be seen in the pruned tree schematic.

When the user selects an element in the overview (by hovering over it with the mouse pointer), the pruned tree is updated to reflect the hierarchy of elements from the root to that currently selected element. The display is "pruned" because it does not show the whole structure, but instead only displays the direct path from the root to the parent that contains the selected element. That is, the pruned tree enables the inspection of all the components directly included in the parent.

**Figure 4:**
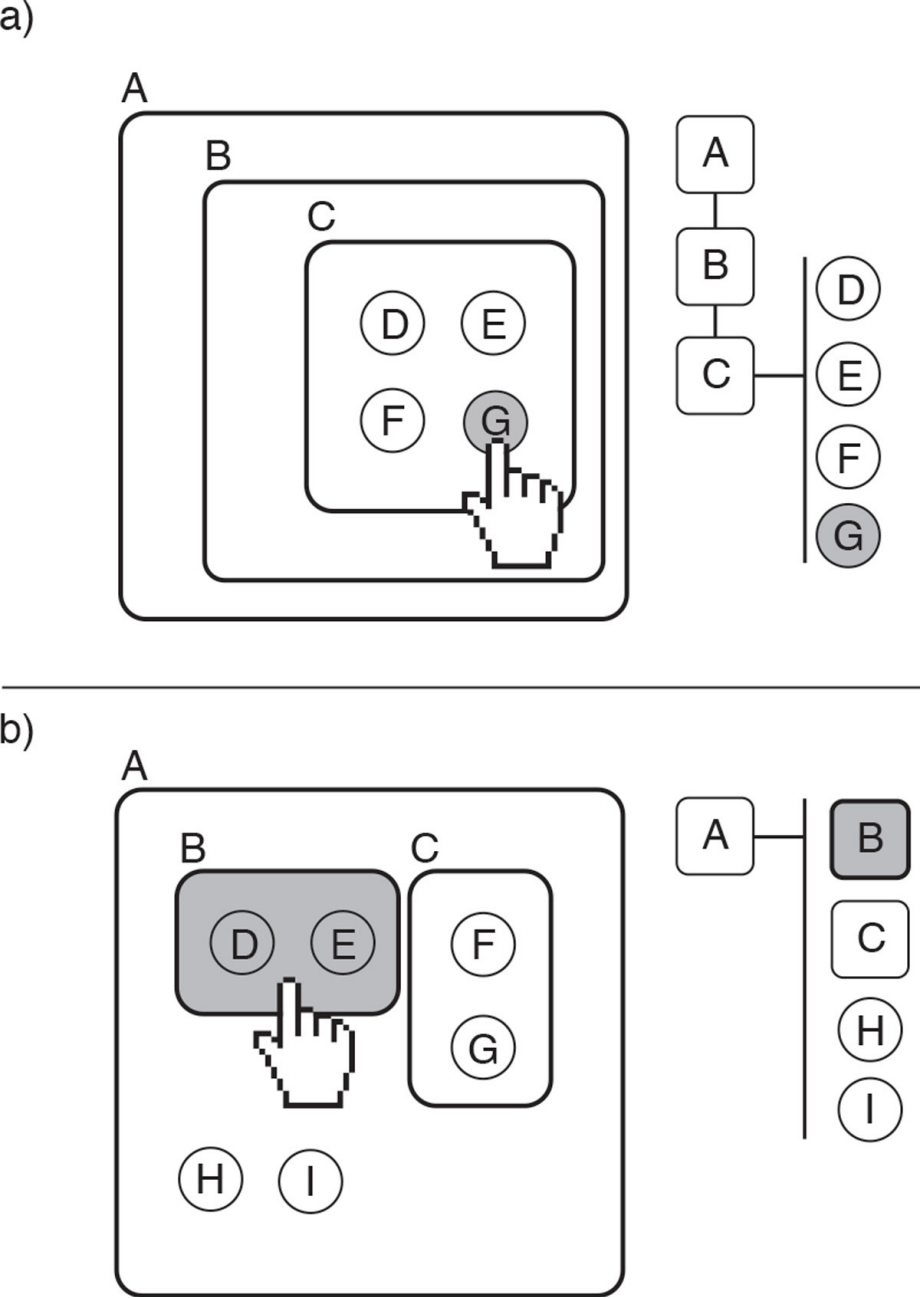
**The figure demonstrates hierarchical inspection using the symbolic overview and the the pruned tree**. In (a), the user selects protein *G *from the expanded node within the node-link diagram; this protein is highlighted within the pruned tree visualization, which also provides more information about its siblings (the proteins *D, E*, and *F*) and its parent and grandparent nodes (the complexes *C, B*, and *A*). In (b), the user selects a complex *B *in the expanded node, which is displayed in a similar manner in the pruned tree.

Figure 4 illustrates how the symbolic overview and the pruned tree are coordinated in order to enable the inspection of a hierarchical structure. In Figure 4(a), the user selects protein *G*, which brings up a pruned tree that indicates the hierarchy of parent complexes that contain *G*, fist *A*, then *B*, and then the direct parent *C*. The right side of the pruned tree shows protein *G *and its siblings *D, E *and *F*. Figure 4(b) illustrates a similar case for complex *B *and its corresponding pruned tree.

**Figure 5:**
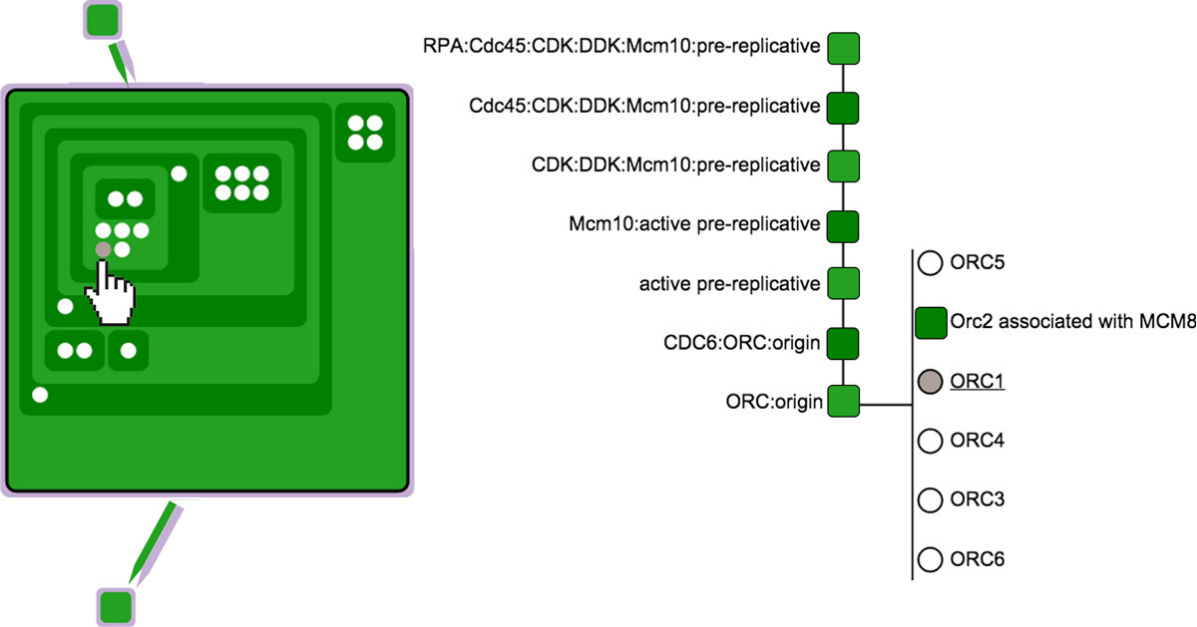
**This figure shows an example of a user inspecting the structure of a protein complex by means of the two coordinated views, the symbolic overview (left) and the pruned tree (right)**.

The symbolic overview provides a compact overview of the structure of the complex, where the squares represent complexes and the white dots are proteins contained within those complexes. Figure 5 shows a child element selected via the mouse pointer. This protein changes its color to gray, and a pruned tree pops up on the display, providing details about this protein and its siblings within the parent complex, as described in the preceding paragraph. By inspecting the pruned tree in Figure 5, the reader can easily read the name of the parent complex of the selected protein, *ORC:origin*, and the related complex and five proteins. For clarity, the hovered component in the symbolic view is underlined and emphasized in the pruned tree. This visual component is integrated in the node-link component previously described. For this reason, the color palette used to differentiate nested complexes is limited to one color hue and two different color intensities, as this minimizes the potential conflict with other colors used to visualize biological elements. Our technique aims to find an optimal balance between reducing the complexity of the displayed structure while at the same providing as much information as necessary for a particular task.

### The prototype application

We created a prototype application that implements our *Extended LineSets *technique. It enables the user to narrow down a potentially large set of proteins and complexes contained within multiple pathways to an arbitrary small group of relevant components. That is, a biologist or bioinformatician can interactively expand or reduce the visible pathways and components that is relevant for his or her research. The prototype uses a force-directed layout [25] to help organize the nodes on the screen. It is not necessarily meant to replace the standard techniques for the visualization of biological pathways, but to offer a novel way for exploring and understanding the relationships within and among pathways. The prototype is accessible online (along with the open source code) at https://github.com/CreativeCodingLab/pathways. By default, for demonstration purposes, the prototype loads in six pathways related to the *Cell Cycle *and 80 sub-pathways. The pathways are referred to as the *field of interest*. The field of interest is comprised of the pathways that the biologist or bioinformatician considers relevant for his or her research. The field of interest initially needs to contain all of the pathways that might be required for the analysis task.

We designed a set of user interactions for exploring, extending and inspecting the pathways at different level of detail. The interaction tasks, discussed below, include the following:

• Pathway highlighting and labelling;

• Pathway hiding and unhiding;

• Keyword filtering;

• Upstream and downstream expansion;

• Layout rearranging, zooming, and panning;

• Complex structure inspection;

• Finding intermediate steps between two nodes in a pathway.

### Pathway highlighting and labeling

**Figure 6:**
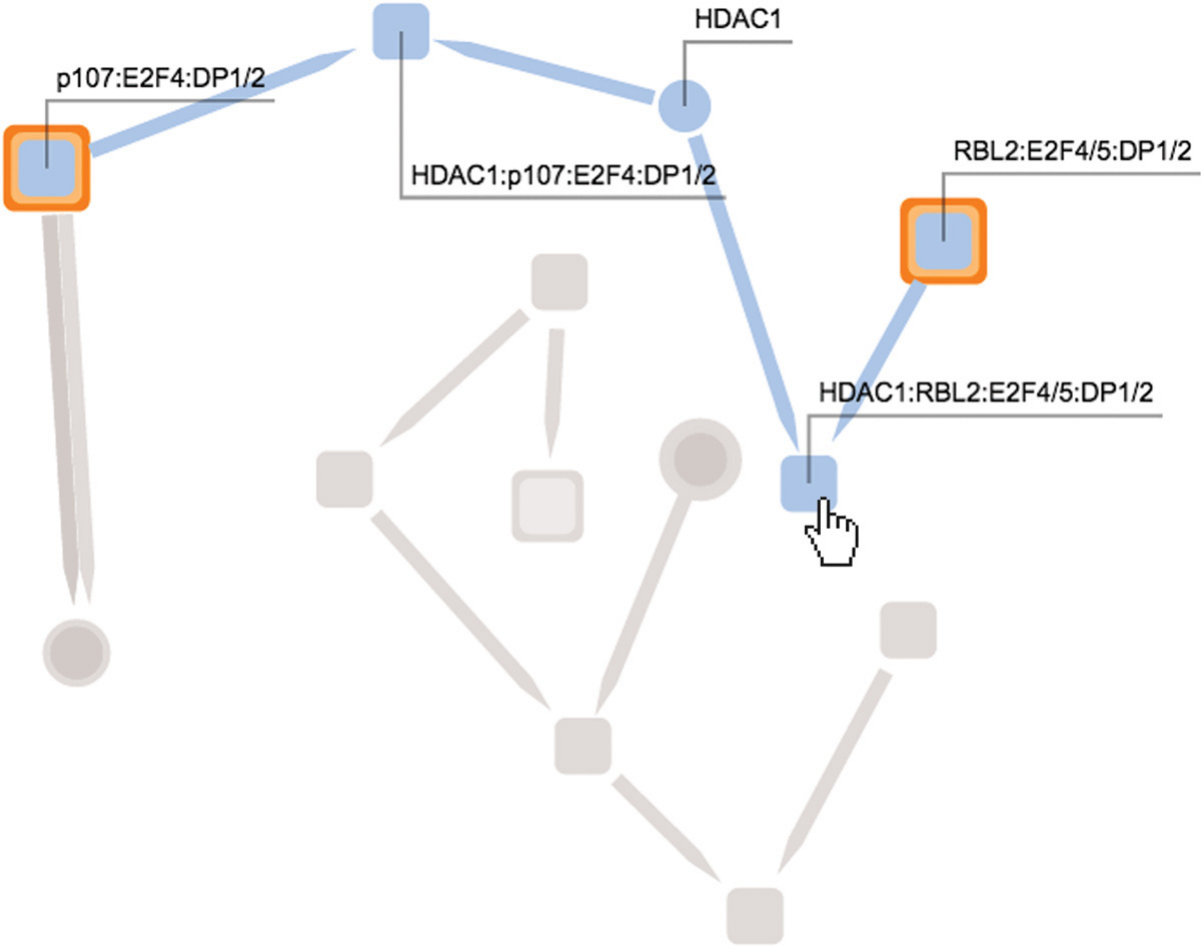
**This figure shows a user hovering over a node, causing the application to highlight only the nodes and links that are members of the same pathways as the selected node**. In this case the node is a protein complex that belongs to a single pathway (indicated by a light blue color). Each element in this pathway (five nodes and four links) are highlighted, while all other elements in the network are grayed-out.

Effective labeling of biological components is required for *Task 1 *and *Task 3*. By default, labels for all complexes and proteins are hidden. This reduces the potential clutter that might be introduced by having too many overlapping labels shown in the graphical representation. Instead, when the user hovers over a component with the mouse pointer, only the labels of the components which are included in the same pathway as the selected component are displayed. The pathways which include this component also stand out because components and reactions belonging to any other pathway are temporarily desaturated (see Figure 6).

### Pathway hiding and unhiding

**Figure 7:**
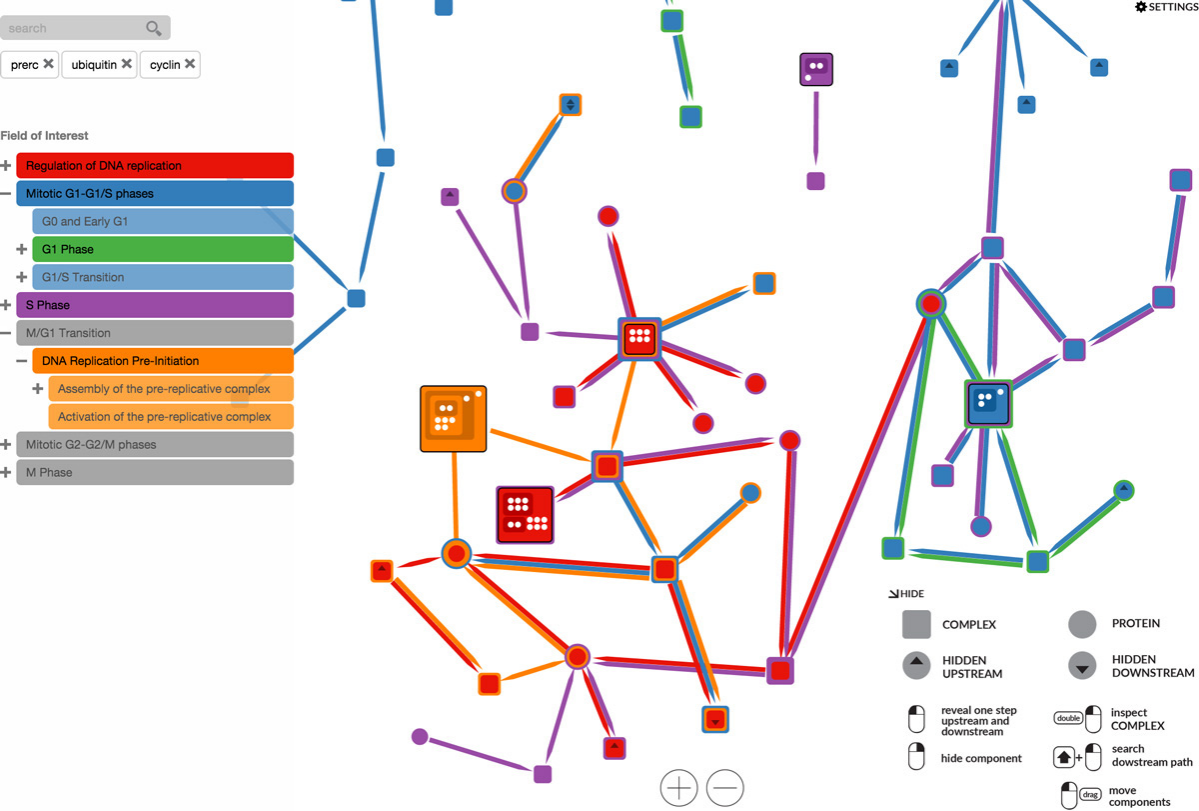
**This figure depicts the interface of our prototype application**. The left side shows, from the top: the search field, the list of searched keywords, and the list of pathways in the field of interest. The right side shows, from the top: a expandable settings menu, and an application guide. In the middle we see the elements of the interconnected pathways that the user is currently investigating.

In order to selectively merge pathway information from different sources (*Task 3*), the user can hide or reveal any pathway included in the field of interest by clicking on its name from the list on the left of the screen (see Figure 7). A given pathway is *visible *if belongs to the field of interest and is not hidden. When a pathway is hidden, its name is colored gray and the visualization is updated to show only the components which are included in at least one visible pathway. Furthermore, the user can delve into the hierarchical structure of sub-pathways by expanding and collapsing the names in the list of pathways.

### Keyword filtering

*Task 3 *requires the ability to create "personalized pathways" on demand. The user can easily add or remove keywords that the system uses to match biological elements. The visualization will display only the proteins (or the complexes containing proteins) whose name matches at least one of the searched keywords.

### Upstream and downstream expansion

By clicking on a protein or complexes the tool will expand the visualization with one *upstream step *and one *downstream step*, starting from the selected component. A downstream step of one protein or complex is composed of all the reaction relationships that start from the given component. Similarly, an upstream step involves all the reaction relationships that end at that component. To facilitate this operation, we place a triangle-shaped maker pointing upwards on the node if it has an hidden upstream step, and a triangle-shaped maker pointing downwards on the node if it has a hidden downstream step. This interaction technique enables the following workflow to support both *Task 1 *and *Task 3 *: first the user searches for a set of proteins and complexes, then he or she progressively explores the network of interconnections by revealing the upstream and downstream nodes.

### Layout rearranging, zooming and panning

Considering that the number of complexes and proteins involved might lead the visualization to exceed the boundary of the screen, the user can interactively zoom in, zoom out, and pan across the viewport. Since the representation might occasionally lead to unavoidable edge intersections, the user can also drag and drop any component on a preferred location to improve viewability.

### Complex structure inspection

In order to support the user's understanding of the hierarchical structure of protein complexes (*Task 2*), our prototype enables the hierarchical inspection of complex structures, as described earlier in this paper. A complex can be enlarged by double-clicking on it. This enlarged complex then reveals its inner structure; all the other components are pushed away to avoid unwanted overlapping. The pruned tree is updated on the right side of the screen when the user moves the mouse over different parts of the hierarchy structure. A single click on an enlarged complex will hide the hierarchical structure, causing it to shrink the complex to the default size. More than one complex can be enlarged at the same time, enabling the user to easily jump from the inspection of one complex structure to another, and to find the same sub-complex in multiple complexes. Indeed, when the user moves the mouse on a component inside a complex, the same component will be highlighted in all the enlarged complexes that contain it.

### Finding intermediate steps between two nodes in a pathway

**Figure 8:**
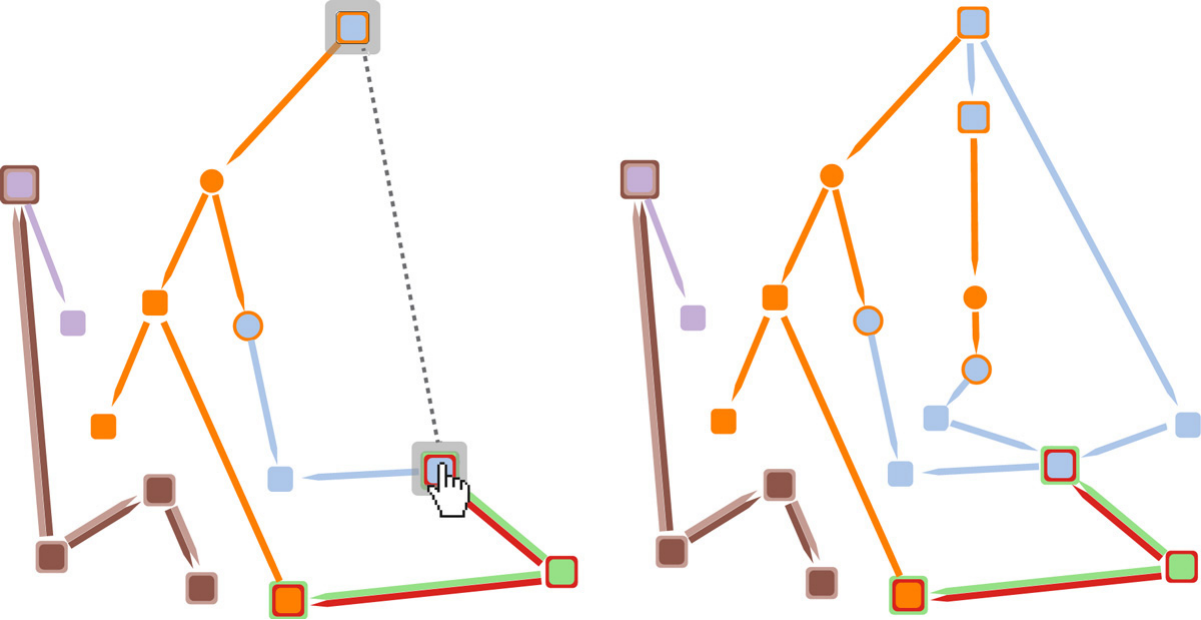
**On the left, the user is in the process of dragging the mouse from one complex to another to indicate to the application that he or she wants to see all the intermediate components that connect these two nodes**. On the right, we see the visualization after it has been updated to include the addition of these intermediate nodes and links.

In addition to the upstream and downstream expansion capabilities previously described, the user can also expand multiple visible components and reaction relationships more quickly. Dragging the mouse while holding right button from one component to another will update the visual representation with *all *of the reaction relationships that start from the first component and end with the last component (as shown in Figure 8). The reaction relationships that are revealed belong only to currently visible pathways. This type of interaction has been designed to help the researcher to create "personalized pathways" that contains only a subset of biological components of interest, which is helpful for *Task 3*.

## Results and discussion

We worked closely with domain experts to verify that our prototype tool provides functionality to enable the tasks we identified in the *Task Analysis *subsection of Section 2. Below we present two use cases to illustrate possible workflows enabled by our application, allowing a user to inspect and interact with a subset of entities and reactions within a biological pathway. We also report expert feedback from two biologists who gave us detailed comments regarding our prototype.

### Exploring the role of ORC1-6 in assembly of the pre-replication complex

In this use case we explore some of the interactions involved in the assembly of the *pre-replication *complex *(preRC) *[26]. We pre-load the prototype application with six pathways involved in the cell cycle. The user expands the menu on the left to view the subpathways of the *M/G1 Transition *pathway and activates the *Assembly of the pre-replicative complex (As-preRC) *and *Activation of the pre-replicative complex (Ac-preRC) *subpathways. The user then searches for *ORC1-6 *and *preRC *in the text field. The visualization is updated with the six *ORC *proteins and the *preRC *complex that match the keywords entered by the user. The color coding shows that the *ORC *proteins are involved in the *As-preRC *pathway, whereas *preRC *in involved in both *As-preRC *and *Ac-preRC*.

The user then interacts with the prototype application to reveal the intermediate steps from the *ORC *proteins to the *pre-replication *complex. Furthermore, the user expands the network to view the immediate downstream elements of *preRC*, revealing reactions involved in the *Ac-preRC *pathway, such as *Mcm10:preRC*, which is expanded again to show the upstream *MCM10 *protein and the downstream *Mcm10:active preRC *protein.

**Figure 9:**
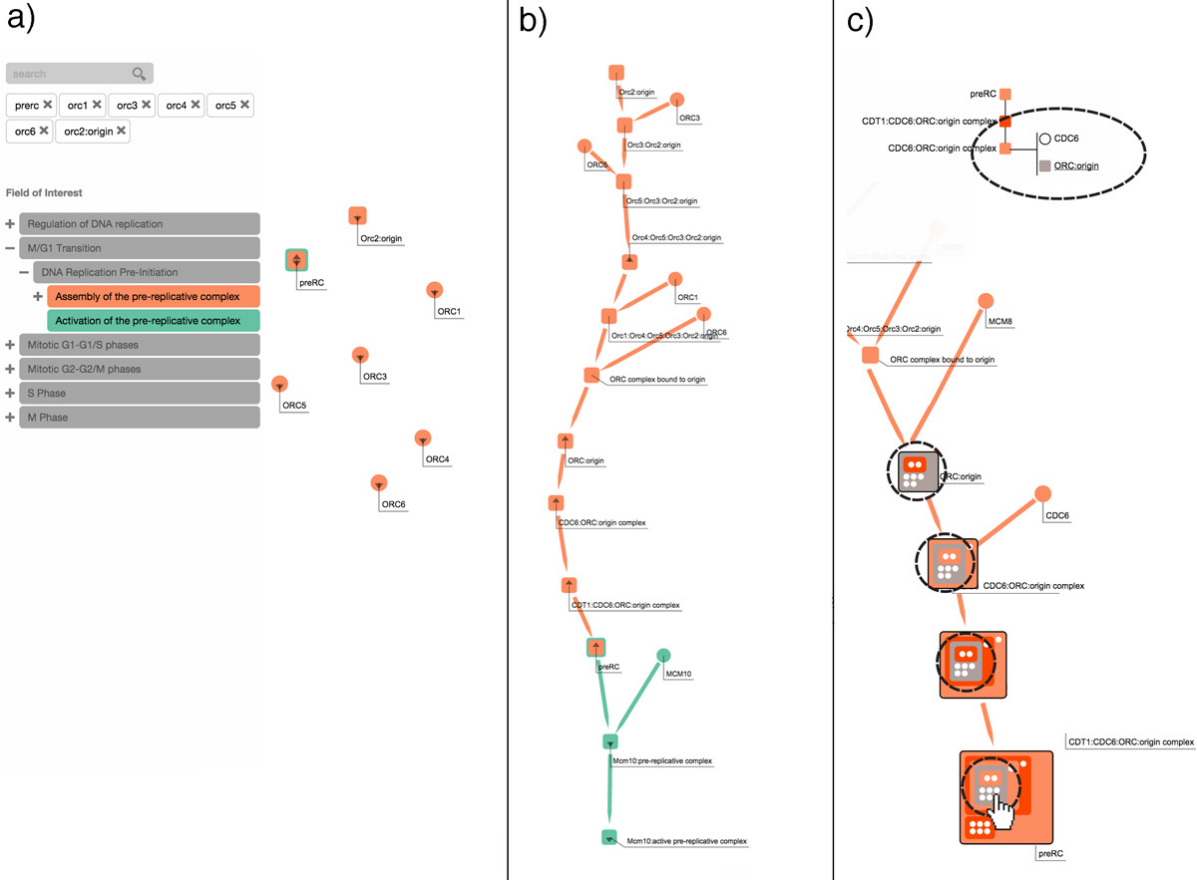
**This figure shows a sequence of user interactions from the first use case, *Exploring the role of ORC1-6 in assembly of the pre-replication complex***. In (a), the user selects two pathways that he or she knows to be interconnected; filtering the pathways via a set of six keywords. In (b), the user interactively expands the relevant nodes, and examines the interconnections between a node in one pathway and another node in another pathway. In (c), the user inspects the hierarchical structure of different complexes.

The user then expands the upstream of *ORC:origin*, revealing the *MCM8 *protein, and expands the downstream complex (*CDC6:ORC:origin*), revealing the upstream protein *CDC6*. Then the user chooses to inspect the hierarchical structure of *preRC, ORC:origin*, along with other two complexes. By hovering over a sub-complex of *preRC*, the user can see that these same sub-complexes appear in all the visible complex structures (since our application automatically highlights them). This enables the user user to better understand the steps involved in the *preRC *assembly. This use case is depicted via the three screenshots shown in Figures 9a-c.

### Exploring pathway interconnections in the cell cycle

In this use case, the user selects three pathways from the larger set of pre-loaded pathways: *Regulation of DNA replication (RDR), M-G1 Transition (MG1) *and *Miotic G1-G1/S phases (G1S)*. The user types into the textbox to search for the *preRC *complex and for proteins that match the *ORC3 *and *Ubiquitin *keywords. By selecting two nodes and dragging between them the user is able to explore the missing steps between, first, *ORC3 *and *preRC *and, second, between *Ubiquitin *and *preRC*. The user can then easily detect which proteins and complexes are involved in all three pathways, such as the *DNA replication factor (Cdt1) *and the *Mini chromosome maintenance complex (MCM2-7) *complex [27].

**Figure 10:**
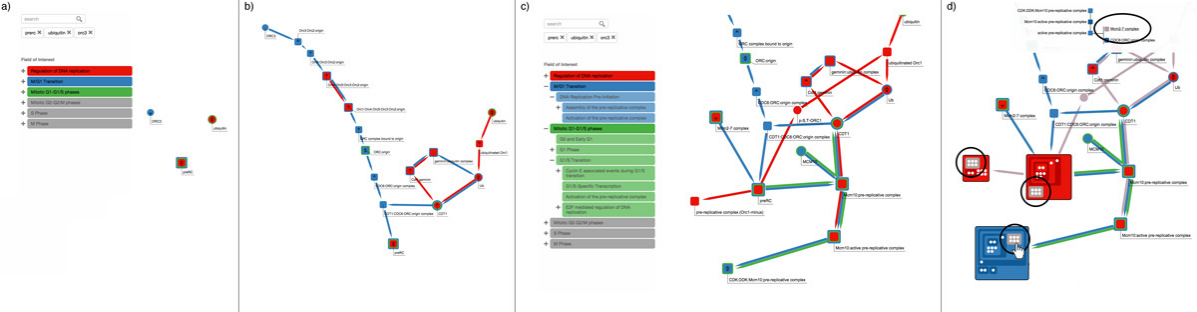
**This figure shows a sequence of user interactions from the second use case, *Exploring pathway interconnections in the cell cycle***. In (a), the user selects two pathways that he or she knows to be interconnected; filtering the pathways via a set of six keywords. In (b), the user interactively expands the relevant nodes, and examines the interconnections between a node in one pathway and another node in another pathway. In (c), the user decides to further investigate subpathways related to *Miotic G1-G1/S phases (G1S)*. In (d), the user inspects the hierarchical structure of different complexes.

Using the integrated visualization of the hierarchy of elements within protein complexes, the user can further inspect the structure of *preRC *and the other complexes, showing, for example, the location within the hierarchical structure of *MCM2-7 *and its six related polypeptides in all the expanded complexes. This use case is explained via the four screenshots shown in Figures 10a-d.

### Expert feedback

We received detailed feedback from two domain experts who confirmed that the design choices implemented in our prototype were effective for pathway analysis tasks. Both of the experts are professors in a biology department at a large public research university. Each of them told us that the ability to see elements shared across multiple pathways (via the different colors) is a very important feature of our prototype. Both experts found the integration of the hierarchical views within the network to be a novel and potentially useful feature. They also appreciated the two coordinated views (the overview within the expanded node and the pruned tree) for inspecting the hierarchical structure of biological complexes.

Additionally, we received positive feedback regarding the search-by-keyword functionality, and especially the step-by-step exploration of the network interconnections. One of the experts agreed that in some cases a more natural way of exploring the network is to first search for a set of known entities and then to freely explore the interconnections to their neighbors. He was especially excited by the fact that these neighbors could be located in different *BioPAX *files or different databases. He also said that the ability to quickly reveal all of the steps between two nodes could be useful for his research into interconnected pathways.

One expert argued that some direct labeling of the nodes would be necessary, at least as an option, as an alternative to the dynamic labelling described earlier. Furthermore, he asked for the possibility to interactively dim all the other pathways to focus on a single pathway. The other domain expert commented that it would be helpful to be able to display the name or type of the reaction between two nodes, perhaps when a user mouses over the link. Moreover, he suggested that it would be useful to include an "undo" operation for when a particular exploration of the network turned out not to be relevant; rather than starting over, he wanted to rewind his exploration to a previous state. For instance, after a node is expanded and its interconnections are revealed, the user might want to revert this operation and hide all of its neighbors. We plan to incorporate these suggestions in a future implementation.

### Future directions

*Extended LineSets *was developed through an iterative process whereby design choices were informally evaluated through discussions with expert users. While we are for the most part happy with the visual encodings and interaction methods that are currently used, we will continue to experiment with alternative encodings and interactions in future iterations. We plan to conduct a thorough empirical evaluation in order to test the effectiveness of our design choices. Although our technique eliminates node redundancy, multiple edges often originate from the same biological reaction. New interactions and visual paradigms should be designed to better represent this information.

Our prototype will require further work in order to become a full-fledged application for enabling pathway visualization tasks. Currently our tool assumes that all relevant pathways are pre-loaded; but the selection of pathways could instead be dynamically loaded from a public database of pathways. Our prototype already reads the *BioPax 3 *format, and a future improvement will be to integrate our tool with the *Pathway Commons *Web API [3]. When our tool is used to find the intermediate steps between two nodes in a pathway, it reveals all the possible sequences of reaction relationships. This could potentially lead, in certain cases, to a jarring increase in the number of elements displayed in the network, especially when dealing with larger pathways with dense interconnections. Future investigations will include the possibility of choosing only the paths that match specified metrics, or by displaying only the shortest path between any two given nodes.

## Conclusions

*Extended LineSets *is a novel technique that enables the interactive visualization of a network of multiple pathways. It introduces a novel graph representation and an effective interactive interface. The visualization aims to provide a better understanding of the biological components and reactions shared among different pathways. Colors are assigned to pathways for a clear visual encoding. The user can dynamically focus on a limited set of pathways to reduce the amount of information and the number of different colors displayed at the same time. User-driven interactions enable a procedural exploration of the network, with the aim of limiting the visual clutter of the graphical representation and minimizing excessive edge crossings. Furthermore, the dynamic labeling of components allows the user to focus on the pathway topology to further reduce the complexity of the visualization. Finally, the inspection of the hierarchical structure of complexes permits the user to change the level of detail of the representation, enabling the simultaneous display of different abstraction layers. Based on real-world use cases and expert feedback, our initial prototype of *Extended LineSets *has already proven to be an effective representation for a range of tasks common to domain experts in biology and bioinformatics.

## Competing interests

The authors declare that they have no competing interests.

## Authors' contributions

FP and AGF conceived of the interactive visualization technique. FP implemented the prototype application and applied it to the two case studies. FP and AGF drafted, read, and approved the final manuscript.

## Declarations

This publication is supported by the DARPA *Big Mechanism *Program under ARO contract WF911NF-14-1-0395.

This article has been published as part of *BMC Proceedings *Volume 9 Supplement 6, 2015: Proceedings of the 5th Symposium on Biological Data Visualization: Part 2. The full contents of the supplement are available online at http://www.biomedcentral.com/bmcproc/supplements/9/S6.
